# Highly efficient heavy-metal extraction from water with carboxylated graphene nanoflakes†

**DOI:** 10.1039/c8ra00823j

**Published:** 2018-03-20

**Authors:** Martin Rosillo-Lopez, Christoph G. Salzmann

**Affiliations:** Department of Chemistry, University College London 20 Gordon Street London WC1H 0AJ UK c.salzmann@ucl.ac.uk

## Abstract

Heavy metals such a lead or cadmium have a wide range of detrimental and devastating effects on human health. It is therefore of paramount importance to efficiently remove heavy metals from industrial wastewater streams as well as drinking water. Carbon materials, including graphene and graphene oxide (GO), have recently been advocated as efficient sorption materials for heavy metals. We show that highly carboxylated graphene nanoflakes (cx-GNF) outperform nano-graphene oxide (nGO) as well as traditional GO with respect to extracting Fe^2+^, Cu^2+^, Fe^3+^, Cd^2+^ and Pb^2+^ cations from water. The sorption capacity for Pb^2+^, for example, is more than six times greater for the cx-GNF compared to GO which is attributed to the efficient formation of lead carboxylates as well as strong cation–π interactions. The large numbers of carboxylic acid groups as well as the intact graphenic regions of the cx-GNF are therefore responsible for the strong binding of the heavy metal cations. Remarkably, the performance of the as-made cx-GNF can easily compete with previously reported carbon materials that have undergone additional chemical-functionalisation procedures for the purpose of heavy-metal extraction. Furthermore, the recyclability of the cx-GNF material with respect to Pb^2+^ loading is demonstrated as well as the outstanding performance for Pb^2+^ extraction in the presence of excess Ca^2+^ or Mg^2+^ cations which are often present under environmental conditions. Out of all the graphene materials, the cx-GNF therefore show the greatest potential for future application in heavy-metal extraction processes.

## Abstract 2

Carboxylated graphene nanoflakes show great potential for heavy-metal extraction from water.

## Graphical abstract

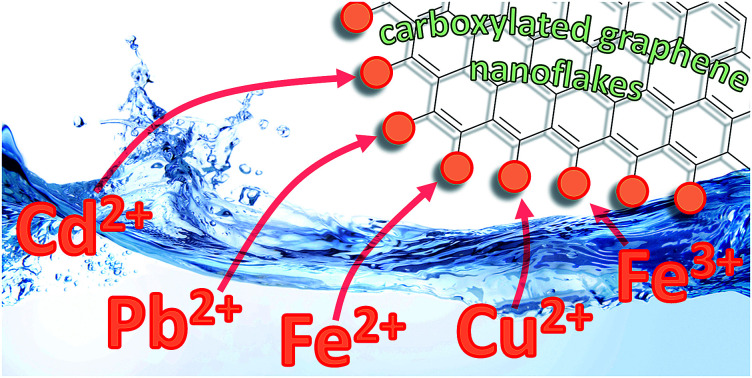

## Introduction

Exposure to heavy metals causes a wide range of adverse health effects in humans.^1^ Lead poisoning, for example, can lead to kidney^2^ and bone damage,^1^ malfunction of the nervous system,^3^ psychosis,^1^ infertility,^4^ anaemia^5^ and cancer.^6,7^ Children in particular are susceptible to the effects of heavy-metal poisoning due to their under-developed blood–brain barrier.^1^ Yet, the global exposure levels of humans to heavy metals are on the rise. This is due, for example, to cadmium-based products such as nickel–cadmium batteries, airborne inorganic lead resulting from mines, smelters, battery plants and the glass industry, and contaminated wastewaters from a wide range of chemical processes in industry.^1,8–10^ The contamination of soil and water streams ultimately leads to the incorporation of heavy metals into the human food chain.^1^ The efficient removal of heavy metals from drinking water, industrial wastewater and the environment at large is therefore of paramount importance.

Carbon materials have been at the vanguard of aqueous heavy-metal extraction over the last few years.^11–31^ This surge in interest arose after the isolation of graphene, a single layer of graphite,^32^ was first employed for metal-extraction processes.^11,12^ More recently, graphene oxide (GO), an oxidised form of graphene containing different functional groups such as epoxides, alcohols and carboxylic acids^33^ has been advocated as an alternative to graphene.^34–36^ The advantage of GO over graphene for metal extraction is its hydrophilic nature, tuneable pore sizes as well as a stronger chelation ability towards metals due to the functional groups. Considerable efforts have gone into chemically modifying GO to further enhance its metal extraction capability including the preparation of hybrid materials with ethylenediamine tetraacetic acid (EDTA),^13,14^ cyclodextrin,^15^ polypyrrole,^16^ polyethylenimine,^17,18^ silica^19^ and many more.^20–23,31^ Recently, some of the functionalised or doped GO materials have been utilised for capacitive-deionisation processes.^26–30^ However, the chemical functionalisation of GO is in general a lengthy and expensive step. It is therefore desirable to optimise and further investigate the metal-extraction properties of as-made carbon nanomaterials in order to provide low-cost adsorbents. In fact, very little research has so far been conducted using nano-graphene oxides (nGO), *i.e.* GO but with lateral dimensions below 100 nm, in heavy metal extraction. This is surprising because the chemical structure of GO, as described by the Lerf–Klinowski model,^33^ suggests that carboxylic acid groups, which should be most effective in chelating metals, are located on the edges of GO. Smaller GO sheets with large edge-to-basal-plane ratios should therefore have more carboxylic acid groups per unit mass and hence be well-suited for metal-extraction processes.

In this work, the metal-sorption capacity of highly edge-carboxylated graphene nanoflakes (cx-GNF)^37^ and nGO^38^ (both ∼30 nm in diameter) are benchmarked against conventional GO prepared *via* the modified Hummers method.^39^ The five heavy-metal cations under investigation are: Fe^2+^, Cu^2+^, Fe^3+^, Cd^2+^ and Pb^2+^. The chemical mechanisms of heavy-metal binding onto the carbon materials and the recyclability of cx-GNF towards loading and unloading of Pb^2+^ cations are investigated as well as the selectivity for Pb^2+^ cation extraction in the presence of large quantities of other cations typically present in drinking or industrial waste water such as magnesium and calcium.

## Experimental

### Preparation of cx-GNF, nGO and GO

The preparation of cx-GNF, nGO and GO is described in detail in ref. 37–39 as well as in Section 1 of the ESI.†

### Adsorption experiments with heavy metals

10 mg of cx-GNF, nGO or GO were combined with 10 mL of 0.1 M solutions of metal chloride or nitrate salts (FeCl_2_·4H_2_O, FeCl_3_·6H_2_O, CuCl_2_·2H_2_O, Cd(NO_3_)_2_·4H_2_O and Pb(NO_3_)_2_). The mixture was then sonicated for 3 × 10 minutes allowing the precipitate to settle out each time for 10 minutes before re-sonicating, resulting in a 60 min total reaction time. This was followed by filtration through a 200 nm polycarbonate membrane and washing with 3 × 10 mL of deionised water to remove any excess metal salt. The material on the membrane was collected and allowed to dry in a vacuum desiccator overnight. The dry powders were analysed by XPS and ATR-IR spectroscopy. For a kinetic study of the adsorption of Pb^2+^ ions onto the carbon materials, the corresponding mixtures were sonicated continuously for a range of different durations up to 100 minutes and worked up as previously described.

### Recyclability of cx-GNF for Pb^2+^ extraction

75 mg of Pb^2+^-loaded cx-GNF (Pb^2+^@cx-GNF) were sonicated in 10 mL of formic acid for <1 min. The dark brown dispersion was diluted with deionised water until pH 2 and then dialysed against deionised water *via* a SpectraPor 3 regenerated cellulose dialysis membrane (Spectrum Laboratories, MWCO 3.5 kDa). Once the conductivity of the surrounding water was below 5 μS cm^−1^ the dispersion was passed over an ion exchange resin (Amberlite IR120, Sigma-Aldrich). The dispersion was dialysed once more, concentrated *in vacuo* and freeze dried to regenerate the cx-GNF. The cx-GNF were then treated again with a 0.1 M Pb^2+^ solution in the same way as previously described.

### Adsorption of Pb^2+^ ions onto cx-GNF in the presence of 10 or 100 molar excess of Ca^2+^ or Mg^2+^ ions

5 mL of a 2 M Ca(NO_3_)_2_ or Mg(NO_3_)_2_ solution was combined with 5 mL of either a 0.2 M or 0.02 M Pb(NO_3_)_2_ solution to give 10 or 100 molar excesses of Ca^2+^ or Mg^2+^ ions. The solutions were then combined with 10 mg of cx-GNF and the precipitated GNFs were collected as described before.

### Adsorption of Pb^2+^ ions onto cx-GNF in commercial drinking water

The ability of the cx-GNF to extract Pb^2+^ cations from commercial drinking water was investigated by preparing a 5 mM solution of Pb(NO_3_)_2_ in Glaceau Smartwater which contains calcium and magnesium chloride as well as potassium bicarbonate. 10 mL of this solution were combined with 10 mg of cx-GNF and the GNFs were collected as described before.

### Sample characterisations

X-ray photoelectron spectroscopy (XPS) measurements were carried out on a Thermo Scientific K-Alpha XPS machine with a monochromated Al Kα source (*E* = 1486.6 eV), a double focusing 180 degree hemisphere analyser of ∼125 mm radius and detected with a 18 channel position-sensitive detector. A dual-beam flood gun (electrons and argon ions) was used to compensate for charge accumulation on the measured surfaces. All carbon samples were pressed onto an indium substrate before analysis. Survey scans were collected 3 times with a resolution of 1 eV and all elemental regions were scanned 10 times with a resolution of 0.1 eV. All scans were recorded with a 50 ms dwell time and 400 μm spot size. All elemental regions were calibrated against the C–C/C=C peak position at 285.0 eV from the C1s high-resolution spectrum. The metal-free cx-GNF data was fitted with two peaks, and a small degree of asymmetry was introduced to the C–C/C=C peak. Yet, the C1s spectra of the M^2+/3+^@cx-GNF were fitted with three peaks.

FT-IR spectra were collected on a Bruker Tensor 27 FTIR spectrometer using the attenuated total-reflectance infrared spectroscopy mode (ATR-IR) fitted with a diamond crystal as the internal reflection element and a DLaTGS detector with 4 cm^−1^ resolution. For each measurement, a background spectrum was collected for 256 scans before recording the sample measurement which was collected for the same number of scans.

High-resolution solid-state ^13^C NMR spectra were recorded on a Bruker Avance 300 spectrometer with a 7.05 T wide-bore magnet at ambient probe temperature. The spectra were recorded at 75.5 MHz with a Bruker 4 mm double-resonance magic-angle spinning (MAS) probe using high-power proton decoupling (HPDEC). For the cx-GNF, the operating conditions were ^13^C 90° pulse duration = 3.7 μs, recycle delay = 120 s with 20 480 scans. Similarly, the operating conditions for the Pb-GNF were ^13^C 90° pulse duration = 3.0 μs, recycle delay = 120 s with 22 528 scans. The cx-GNF and Pb^2+^@cx-GNF were packed into zirconia rotors of 4 mm external diameter and spun at 12 kHz or 8 kHz MAS frequency respectively. In each case tetramethylsilane (TMS) was calibrated against an aqueous solution of 4,4-dimethyl-4-silapentane-1-sulfonic acid (DSS, 0 ppm) and glycine (176.46 ppm), and the ^13^C chemical shifts are reported with respect to TMS.

### Determination of Pb^2+^ sorption capacities with optical absorbance spectroscopy

10 mg of cx-GNF, nGO or GO were combined with 10 mL of 5 mM Pb(NO_3_)_2_ solutions. The mixtures were then ultrasonicated, filtered and washed as previously described. The colourless filtrates as well as 10 mL of a 5 mM Pb^2+^ solution were topped up to 250 mL with deionised water. 4 mL of these solutions were acidified with 1 mL of 5 mM HCl, combined with a solution of 2,5-dimercapto-1,3,4-thiadiazole dipotassium salt (DMTD-K^+^)^40^ and topped up to 10 mL. The yellow solutions were then transferred into quartz cuvettes of 1 cm path length, and optical absorbance spectra were recorded between 300 and 500 nm on a PerkinElmer Lambda 365 spectrophotometer at steps of 1.0 nm and scan rate of 600 nm min^−1^. Finally, the Pb^2+^ sorption capacities of the carbon materials were calculated from the difference in the optical absorbances at 400 nm between the initial 5 mM solution and the solutions after the Pb^2+^ extraction.

## Results and discussion

### Structural characterisation of the cx-GNF, nGO and GO starting materials

**Fig. 1:**
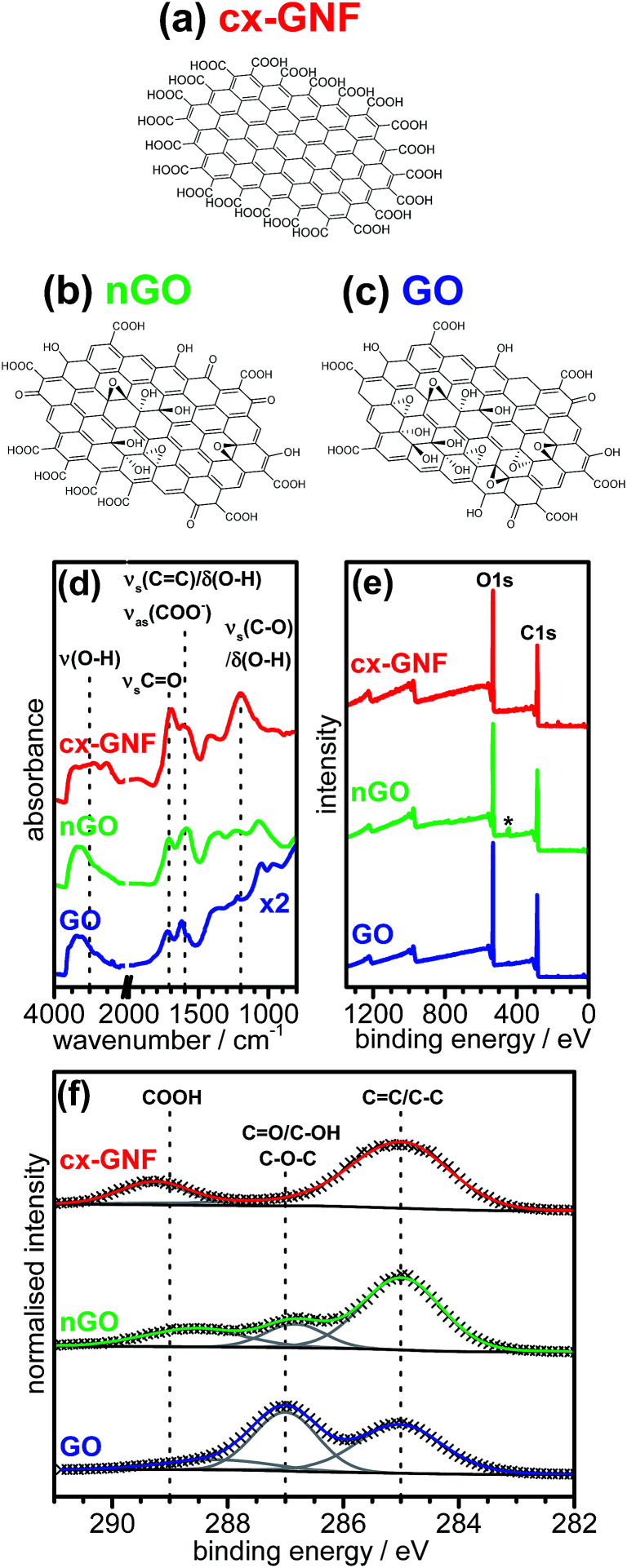
Schematic chemical structures of (a) cx-GNF, (b) nGO and (c) GO. The lateral dimensions of cx-GNF and nGO are approximately 30 nm whereas GO sheets can be up to several μm in diameter. (d) ATR-IR spectra, (e) XPS survey and (f) C1s regions of cx-GNF (red), nGO (green) and GO (blue). The peak marked with the asterisk in (e) is due to the indium substrate.

The chemical structures of cx-GNF, nGO and GO as well as the corresponding ATR-IR and XPS spectra are shown in Fig. 1a–f. The cx-GNF are a highly carboxylated graphenic nanomaterial^37^ as indicated by the very broad O–H stretching frequency peak between 3600 cm^−1^ and 2500 cm^−1^ in the IR spectrum (Fig. 1d). This is further corroborated by a strong peak at 289.3 eV in the XPS C1s region which is associated with C(iii) species such as carboxylic acids (Fig. 1f).^41^ In contrast to the cx-GNF, nGO and GO show the presence of a peak at approximately 287 eV which is attributed to C(i) species such as alcohols and epoxides, and even C(ii) species such as ketones.^42,43^ This peak is significantly more intense for GO than for nGO because these functional groups are typically found on the basal plane of oxidised graphenic materials, and GO has a larger basal-plane-to-edge ratio compared to nGO.^38^ Furthermore, the edges of nGO and GO are only sparingly decorated with carboxylic acids amongst other functional groups,^33^ and since nGO has more edges than GO, on account of nGO being 1–2 orders of magnitude smaller than GO, nGO will have more carboxyl groups than GO per unit mass; as evidenced by the C1s spectrum in Fig. 1f. By analogy, if a piece of paper is cut multiple times, the mass of the paper will remain the same after being cut, yet the overall length of the edges of the smaller cut-out sheets will exceed that of the uncut paper. Finally, for all three carbon materials, the peak attributed to graphenic carbon can be clearly seen at ∼285 eV. Full structural characterisations and investigations into the specific chemical properties of cx-GNF, nGO and GO are given in ref. 37–39 respectively.

### Heavy-metal extraction from water with cx-GNF, nGO and GO

**Fig. 2:**
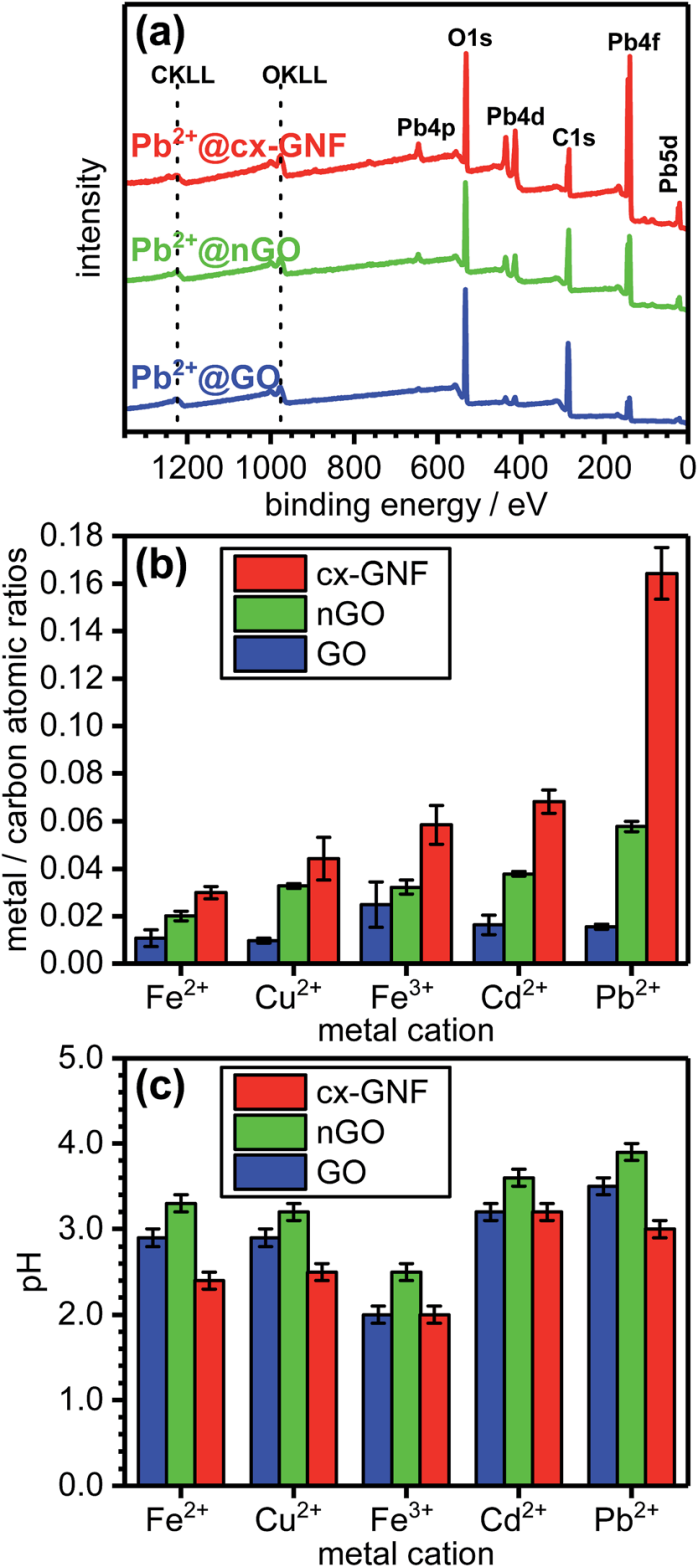
(a) XPS survey spectra of Pb^2+^@cx-GNF (red), Pb^2+^@nGO (green) and Pb^2+^@GO (blue). (b) Metal ion/carbon atomic ratios. (c) pH values of the Fe^2+^, Cu^2+^, Fe^3+^, Cd^2+^ or Pb^2+^ solutions treated with cx-GNF (red), nGO (green) and GO (blue), respectively. The pH of each of the mixtures was measured prior to filtration (10 mg of graphenic material in 10 mL of 0.1 M solutions of the respective metal salts).

In order to quantify the relative sorption capacities of cx-GNF, nGO and GO towards heavy metals, the metal/carbon (M^2+/3+^/C) ratio of each metal–carbon composite, denoted as M^2+/3+^@cx-GNF, M^2+/3+^@nGO or M^2+/3+^@GO, respectively, or collectively as M^2+/3+^@carbon, was determined from the XPS survey spectra. Fig. 2a shows survey spectra of cx-GNF, nGO and GO after exposure to Pb^2+^ solutions. In the case of Pb^2+^@carbon, the Pb4f peak was used for the quantification of Pb^2+^. The XPS survey spectra of the other M^2+/3+^@carbon samples can be found in Fig. S1 of the ESI.† The determined M^2+/3+^/C ratios for the various heavy metals and carbon materials are shown in Fig. 2b. It is also noted that significant mass increases of the carbon materials were observed after some of the heavy-metal extractions. For example, the weight of the cx-GNF material increased by about 30 weight percent after the Pb^2+^ sorption experiment.

The ability of a carbon material to extract metal cations from solution is dependent upon a range of parameters including the ionic strength and pH of the mixture. In order to ensure comparable ionic strengths throughout the various adsorption experiments, 10 mg of each carbon material was treated with 10 mL of a 0.1 M solutions of the metal salt which means that the cations are in large excess. Consequently, the ionic strength of a mixture of cx-GNF, nGO or GO with a particular metal cation, is approximately constant.

Likewise, increasing the pH of a mixture is expected to lead to an increase in metal chelation. This arises as a result of a decrease in H^+^ ions in solution which in turn reduces the competition between the metal cations and H^+^ ions for the chelating ligand; which in the case of cx-GNF, nGO and GO, are the oxygen-containing functional groups. Furthermore, increasing the pH to basic conditions would lead to precipitation of metal hydroxide species^44,45^ and therefore give misleading results.

The pH of the M^2+/3+^@carbon mixtures was measured prior to filtration and the results are shown in Fig. 2c. It can be seen that for a particular metal ion, the pH of the M^2+/3+^@cx-GNF ≤ M^2+/3+^@GO < M^2+/3+^@nGO indicating that the cx-GNF are at a disadvantage for metal sorption with respect to GO and nGO for the reasons discussed earlier. The lower pH of the cx-GNF is expected given the highly carboxylated nature of the material and the fact that carboxylic acids are much stronger acids than alcohols and the other functional groups found at high concentrations on GO. Indeed, 1 mg mL^−1^ aqueous dispersions of cx-GNF, nGO and GO gave pH values of 2.5, 3.3 and 3.0 respectively. Interestingly, the pH of GO is slightly lower than nGO despite nGO containing more carboxylic acid groups. However, GO has many more acidic hydroxyl groups on its basal plane, and given that its structural instability in water generates even more acidic functional groups,^46^ this result is not necessarily surprising.

Despite the lower pH of the cx-GNF, they clearly outperform the other two carbon materials at extracting metals as evidenced by the M^2+/3+^/C ratios obtained from the XPS survey spectra shown in Fig. 2b. For example, the cx-GNF can bind about 10 times more Pb^2+^ than GO. This can be rationalised at this stage by consideration of the number of carboxylic acid groups per unit mass of each material. According to peak–area ratios from solid-state NMR measurements (COOH/graphenic carbon),^37,38^ the cx-GNF contain about four times more COOH groups than nGO, and about 10 times more COOH groups than GO.

Carboxylic acids are bidentate and generally stronger metal chelators compared to monodentate alcohols and epoxides. Indeed, it has been previously stated that COOH groups are the most efficient functional group present on graphenic materials for chelating Pb^2+^ cations.^11^ It is important to stress that despite nGO achieving better extraction results compared to GO, the pH of the M^2+/3+^@nGO mixtures were on average 0.4 units more basic than for M^2+/3+^@GO, allowing the nGO flakes to better coordinate to metals. Hence, the actual ability of nGO to remove heavy metals compared to GO may not be as pronounced as shown in Fig. 2b if the extractions were carried out at the same pH.

It is noteworthy that we deliberately did not use buffer solutions for these experiments and relied on the natural pH of the M^2+/3+^@carbon mixtures. This removes any doubt with respect to the effect ionic strength and foreign species might have in influencing the metal-sorption capability of the carbon materials.

A kinetic study of the Pb^2+^ adsorption onto the various carbon materials shows that the adsorption equilibria are reached very quickly for nGO and GO (Fig. S2 of the ESI†). The cx-GNF show a slight gradual increase in the Pb^2+^ uptake over a time period of 100 minutes. This could be due to the initial formation of dicarboxylate–lead complexes with COOH groups from different flakes which are successively replaced by monocarboxylate complexes as the sonication time progresses.

The fast equilibration of the Pb^2+^ adsorption onto nGO and GO illustrates that their poorer performance with respect to binding Pb^2+^ is not due to kinetic factors but to the intrinsically different interaction properties of the carbon materials with the heavy metals. Furthermore, the exfoliation process of the carbon materials upon sonication, which could potentially limit the accessible surface area, does not appear to be a limiting factor for the heavy metal extraction using nGO and GO.

**Fig. 3:**
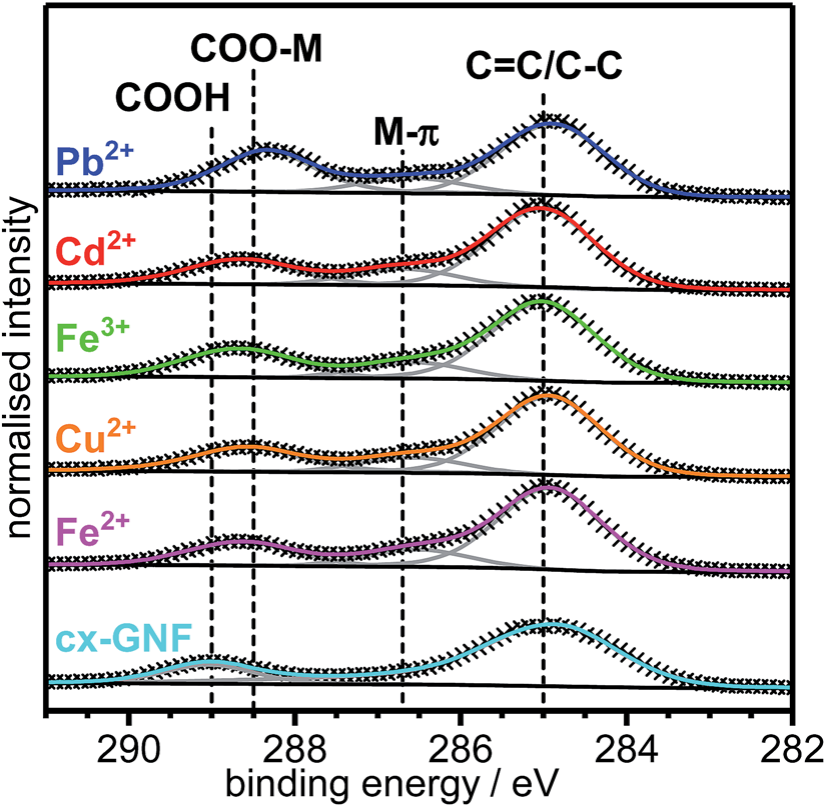
Area-normalised XPS C1s spectra of cx-GNF (cyan), Fe^2+^@cx-GNF (magenta), Cu^2+^@cx-GNF (orange), Fe^3+^@cx-GNF (green), Cd^2+^@cx-GNF (red) and Pb^2+^@cx-GNF (blue).

The area-normalised C1s XPS spectra of the M^2+/3+^@cx-GNF are shown in Fig. 3. The most immediate observation are additional peaks at ∼286.7 eV which are present in all the M^2+/3+^@cx-GNF samples but absent for the pure cx-GNF. Furthermore, the areas of the C(iii) peaks at ∼288.5 eV are larger and shifted towards lower binding energies for the M^2+/3+^@cx-GNF. The newly formed peaks at 286.7 eV suggest non-covalent cation–π interactions between the metal cations and the graphenic basal plane of the cx-GNF.^47–49^ This can be rationalised by considering the electrostatic attraction between the positively charged metal cations and the graphenic π-electrons which creates a local deshielding effect along the graphenic basal plane where the metal cations have adsorbed. This in turn confers a *δ*^+^ charge on the carbon atoms resulting in an increase in the binding energy of its core electrons. Hence, the observed decrease in the relative C1s C–C/C=C peak intensity with respect to the C(iii) peak. This effect is most noticeable for the Pb^2+^@cx-GNF sample which has the highest M^2+/3+^/C ratio of all the M^2+/3+^@cx-GNF (Fig. 2b), suggesting that Pb–π interactions are the most favourable out of all the heavy metals under these conditions, perhaps because Pb^2+^ has the lowest solvation enthalpy.^50^

**Fig. 4:**
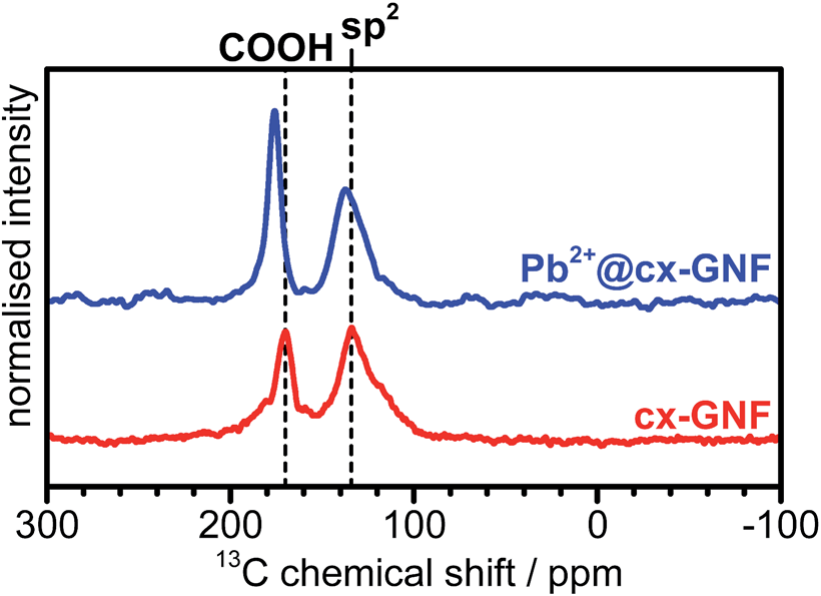
^13^C-NMR spectra of cx-GNF (red) and Pb^2+^@cx-GNF (blue). Vertical dashed lines denote peak positions of functional groups.

To further prove that the trends observed in the XPS C1s spectra are due to cation–π interactions, solid state ^13^C-NMR spectra of the cx-GNF were collected before and after exposure to a Pb^2+^ solution (Fig. 4). The ^13^C-NMR spectra of the cx-GNF and Pb^2+^@cx-GNF both exhibit two peaks, one associated with COOH groups (∼170 ppm) and another for sp^2^ graphenic carbon (∼130 ppm).^33^ A distinct shift to higher ppm (downfield shift) is observed for both peaks in the spectrum of Pb^2+^@cxGNF, indicating more deshielded environments. The downfield shift observed for the sp^2^ carbon from 134 to 137 ppm is in good agreement with M–π interactions which induce a deshielding effect on the aromatic rings as described earlier.^48^ However, the downfield shift of the COOH group from 170 to 176 ppm is most likely the result of chelation between the Pb^2+^ ions and the carboxyl groups on the cx-GNF.^51,52^ It is noteworthy that the absence of C(i) species such as epoxides and alcohols between 60 and 70 ppm in the Pb^2+^@cx-GNF spectrum proves that the newly formed peaks at 286.7 eV in the XPS C1s regions of the M^2+/3+^@cx-GNF are due to M–π interactions and not to the formation of alcohol or epoxide groups. Interestingly, the M–π interactions were not observed in the XPS C1s spectra of M^2+/3+^@GO but were noticeable to a small extent for the M^2+/3+^@nGO materials (Fig. S3†). This could be because nGO nanomaterials have more intact aromatic sp^2^ basal planes compared to GO, as evidenced by the higher intensity of the sp^2^ C=C peak in the solid state ^13^C-NMR spectra.^37,38^

**Fig. 5:**
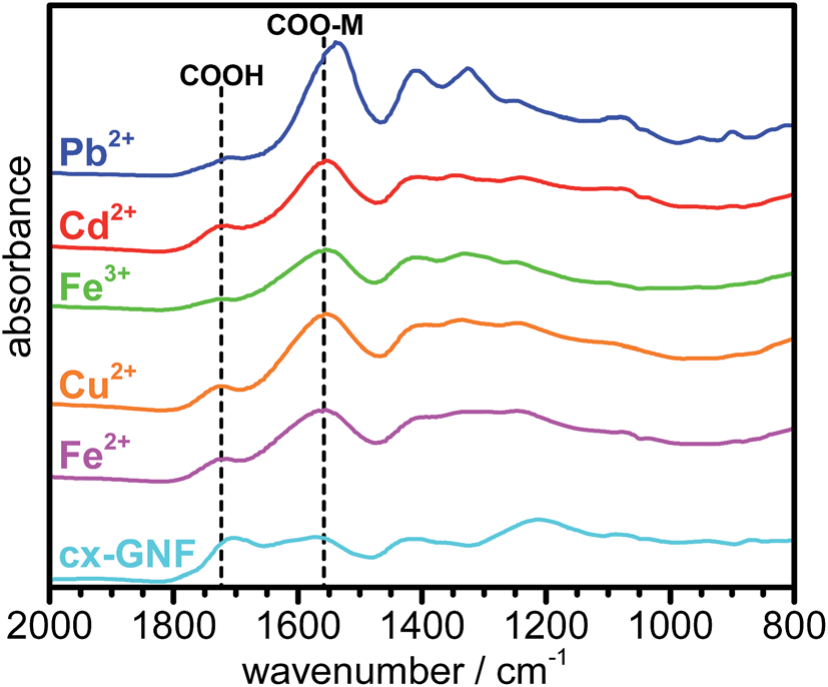
ATR-IR spectra of cx-GNF (cyan), Fe^2+^@cx-GNF (magenta), Cu^2+^@cx-GNF (orange), Fe^3+^@cx-GNF (green), Cd^2+^@cx-GNF (red) and Pb^2+^@cx-GNF (blue).

Further evidence for metal chelation between the heavy-metal cations and the carboxylic acid groups of the cx-GNF is provided by the significant shift to lower binding energy of the XPS C1s C(iii) peak from ∼289 eV to 288.5 eV (Fig. 3), consistent with the weakening of the C=O bond in COOH to form an extended electron delocalised system in the metal carboxylate.^53^ This is further emphasized by the bathochromic shift in the C=O stretching frequency from about 1715 cm^−1^ of the COOH groups in cx-GNF to 1558 cm^−1^ of the metal carboxylates in the M^2+/3+^@cx-GNF samples (Fig. 5). Interestingly, the effect is also most pronounced for Pb^2+^@cx-GNF, suggesting that Pb^2+^ ions interact most strongly with the cx-GNF both *via* M–π interactions as well as the metal–carboxylate chelation. Consequently, these two effects taken together explain the exceptionally high performance of the cx-GNF for extraction of Pb^2+^ compared with the other carbon materials as shown in Fig. 2b. It is noteworthy that the IR spectra of both the M^2+/3+^@nGO as well as M^2+/3+^@GO also show the same bathochromic shift (Fig. S4†) indicating that metal complexation has taken place, albeit to lesser extents.

### Reversible loading and unloading of Pb^2+^ ions onto cx-GNF

**Fig. 6:**
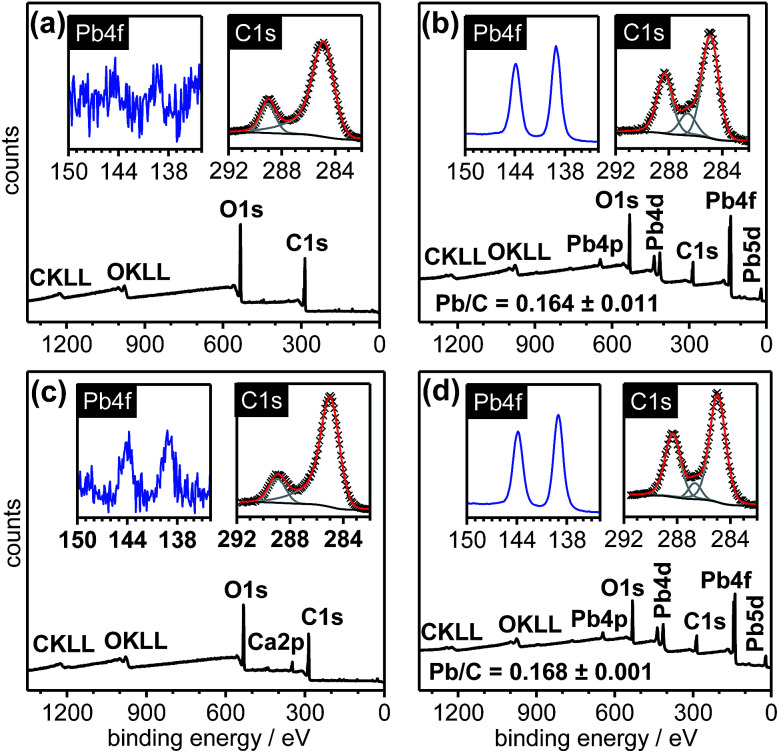
Reversible loading of Pb^2+^ ions onto cx-GNF. XPS survey spectra (outset), Pb4f regions (inset, left) and C1s regions (inset, right) of (a) cx-GNF before Pb^2+^ addition, (b) after Pb^2+^ addition, (c) treatment of (b) with formic acid to remove Pb^2+^ and (d) after reloading with Pb^2+^.

The XPS spectra in Fig. 6 illustrate the reversible loading and unloading of Pb^2+^ ions onto the cx-GNF. Initially, the cx-GNF (Fig. 6a) were treated with 0.1 M Pb^2+^ solutions in the same way as described earlier yielding the corresponding Pb^2+^@cx-GNF materials (Fig. 6b). The Pb^2+^ ions were then removed by treatment with formic acid (Fig. 6c), leaving behind only a small trace of Pb^2+^ ions. A small quantity of calcium (3%) was also detected in the XPS survey spectrum which was most likely the result of the gradual uptake of Ca^2+^ ion traces in the deionised water during the multiple dialysis steps to remove the Pb^2+^ ions (see Experimental section). It is noteworthy that after the unloading of Pb^2+^ ions from the Pb^2+^@cx-GNF there is clear reversibility in the XPS C1s region back to the original cx-GNF in terms of the relative peak intensities. Finally, after removal of the Pb^2+^ ions, the cx-GNF were re-treated with 0.1 M Pb^2+^ solution as before which again yielded Pb^2+^@cx-GNF without any change in the loading efficiency as indicated by the Pb^2+^/C ratio (Fig. 6d). As expected due to the reversible behaviour, the final XPS C1s region was similar compared to the spectrum of the Pb^2+^@cx-GNF upon first loading of the cx-GNF with Pb^2+^ ions.

### Selectivity of cx-GNF towards Pb^2+^ cations in the presence of 10 and 100 molar equivalents of Ca^2+^ and Mg^2+^ cations

**Fig. 7:**
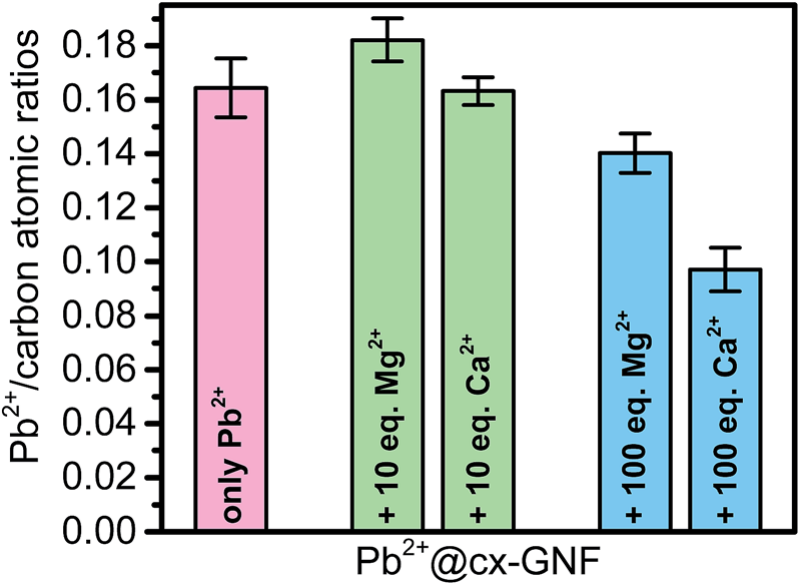
Pb^2+^/C atomic ratios determined from XPS survey spectra of cx-GNF treated with Pb^2+^ only (magenta), Pb^2+^ with 10 equivalents Ca^2+^ or Mg^2+^ ions (green), and Pb^2+^ with 100 equivalents Ca^2+^ or Mg^2+^ ions (blue).

To assess the selectivity of the cx-GNF towards Pb^2+^ extraction in the presence of other cations, the cx-GNF were treated with mixtures containing lead ions as well as calcium or magnesium cations. Calcium and magnesium were chosen because these cations are the most common divalent metal cations found in drinking or industrial waste waters. The Mg^2+^ or Ca^2+^ cations were either in a 10 or 100 molar excess compared to the lead ions. In the presence of 10 mole equivalents of Ca^2+^ or Mg^2+^ ions per equivalent of Pb^2+^, only Pb^2+^ ions were removed from solution (Fig. 7, S5a and c† for XPS spectra) and in the same quantities as reported before in Fig. 2b where no other cations were present. When 100 mole equivalents were employed, there was still a significant selectivity towards the Pb^2+^ ions as shown in Fig. 7. A Pb^2+^/Mg^2+^ ratio of 6.5 and Pb^2+^/Ca^2+^ ratio of 2.0 was determined from the XPS survey spectra (Fig. S5b and d†).

Upon adding 5 mM Pb(NO_3_)_2_ to commercial drinking water, which contains mainly CaCl_2_, MgCl_2_ and KHCO_3_, it was found that the Pb^2+^ extraction with cx-GNF was not affected by the presence of Mg^2+^ in the drinking water. Small amounts of Ca^2+^ could be identified in the XPS spectra of the cx-GNF material after the extraction process (*cf.* Fig. S6†). Yet, the Pb^2+^/Ca^2+^ atomic ratio was found to be 15 ± 3 illustrating that the presence of typical amounts of alkaline-earth metal ions in drinking water do not overall significantly affect the performance of the cx-GNF with respect to extracting Pb^2+^.

### Determination of Pb^2+^ sorption capacities with optical absorbance spectroscopy

To determine the sorption capacities in milligrams of lead extracted per gram of carbon material, a photometric approach was used as described in the Experimental section. Calibration curves were recorded to ensure the Beer–Lambert law was obeyed at *λ* = 400 nm in the concentration range used in these experiments (Fig. S7†). To avoid unfavourably large absorbance values, more dilute 5 mM Pb(NO_3_)_2_ solutions were used instead of the earlier 0.1 M solutions. However, using XPS it was shown that the Pb^2+^/C atomic ratios of the Pb^2+^@carbon materials were the same regardless of whether 5 mM or 0.1 M Pb^2+^ solutions were used (Fig. S8† and 2b). This means that quantitative loading of the carbon materials with Pb^2+^ is achieved at both concentrations.

The sorption capacities of the cx-GNF, nGO and GO for Pb^2+^ were calculated from the difference in the optical absorbances at 400 nm between the initial 5 mM solution and the solutions after the Pb^2+^ extraction (Fig. S9†); and found to be 659, 336 and 102 mg g^−1^, respectively, at pH values of the solutions of 2.2, 3.3 and 3.1. The Pb^2+^ sorption capacity of the cx-GNF is therefore more than six times that of conventional GO. Again, we note that under identical pH conditions, this value is likely to be even higher.

In addition to being the best material for Pb^2+^ extraction out of the investigated as-made carbon nanomaterials, the cx-GNFs can easily compete with most of the tailored chemically-functionalised carbon materials in terms of sorption capacity. Reviews of the heavy-metal sorption capacities of a wide range of chemically-modified carbon materials are given in ref. 23 and 31.

Finally, an attempt was made to determine the Pb^2+^ sorption capacity of activated charcoal (AC), but it was found to be negligible in comparison with the graphenic materials investigated here. Indeed, the Pb^2+^/C atomic ratio of Pb^2+^@AC was determined to be one order of magnitude below GO and therefore two orders of magnitude lower than for cx-GNF (Fig. S10†). This is in good agreement with Yang *et al.* who demonstrated the adsorption capacity of Cu^2+^ cations on GO is about ten times higher than activated charcoal.^54^

## Conclusions

Carboxylated graphene nanoflakes are a highly efficient carbon material for the extraction of a wide range of heavy metals from water. They outperform the conventional GO and can easily compete with carbon nanomaterials that have been chemically-functionalised for the purpose of heavy-metal extraction.^23,31^ A remarkable affinity for Pb^2+^ ions even in the presence of large excesses of Mg^2+^ and Ca^2+^ ions has been demonstrated. Detailed insights into the chemical binding mechanisms of the heavy metal cations onto the cx-GNFs were gained highlighting the formation of metal carboxylates as well as M–π interactions as the two dominating modes for metal–carbon interactions. The high efficiency of the cx-GNF for heavy-metal extraction can therefore be attributed to the large number of carboxylic acid groups but also the intact graphenic areas on the basal plane. The loading and unloading of Pb^2+^ ions onto the cx-GNF was found to be completely reversible allowing for the cx-GNF to be readily recycled. Consequently, out of all the investigated as-made graphene materials, the cx-GNF are most suited for future applications in heavy-metal extraction processes.

## Conflicts of interest

There are no conflicts to declare.

## Supplementary Material

RA-008-C8RA00823J-s001

## References

[cit1] Jarup L. (2003). Br. Med. Bull..

[cit2] Mortada W. I., Sobh M. A., El-Defrawy M. M., Farahat S. E. (2001). Am. J. Nephrol..

[cit3] Lidsky T. I., Schneider J. S. (2003). Brain.

[cit4] Apostoli P., Bellini A., Porru S., Bisanti L. (2000). Am. J. Ind. Med..

[cit5] Schwartz J., Landrigan P. J., Baker E. L., Orenstein W. A., Vonlindern I. H. (1990). Am. J. Public Health.

[cit6] Steenland K., Boffetta P. (2000). Am. J. Ind. Med..

[cit7] Silbergeld E. K., Waalkes M., Rice J. M. (2000). Am. J. Ind. Med..

[cit8] Barakat M. A. (2011). Arabian J. Chem..

[cit9] Hua M., Zhang S., Pan B., Zhang W., Lv L., Zhang Q. (2012). J. Hazard. Mater..

[cit10] Hsu L.-C., Huang C.-Y., Chuang Y.-H., Chen H.-W., Chan Y.-T., Teah H. Y., Chen T.-Y., Chang C.-F., Liu Y.-T., Tzou Y.-M. (2016). Sci. Rep..

[cit11] Huang Z. H., Zheng X. Y., Lv W., Wang M., Yang Q. H., Kang F. Y. (2011). Langmuir.

[cit12] Deng X. J., Lu L. L., Li H. W., Luo F. (2010). J. Hazard. Mater..

[cit13] Cui L. M., Wang Y. G., Gao L., Hu L. H., Yan L. G., Wei Q., Du B. (2015). Chem. Eng. J..

[cit14] Madadrang C. J., Kim H. Y., Gao G. H., Wang N., Zhu J., Feng H., Gorring M., Kasner M. L., Hou S. F. (2012). ACS Appl. Mater. Interfaces.

[cit15] Fan L. L., Luo C. N., Sun M., Qiu H. M. (2012). J. Mater. Chem..

[cit16] Li S. K., Lu X. F., Xue Y. P., Lei J. Y., Zheng T., Wang C. (2012). PLoS One.

[cit17] Sui N., Wang L. N., Wu X. H., Li X. H., Sui J., Xiao H. L., Liu M. H., Wan J., Yu W. W. (2015). RSC Adv..

[cit18] Liu Y., Xu L., Liu J. S., Liu X. Y., Chen C. H., Li G. Y., Meng Y. F. (2016). Chem. Eng. J..

[cit19] Sitko R., Zawisza B., Talik E., Janik P., Osoba G., Feist B., Malick E. (2014). Anal. Chim. Acta.

[cit20] Zhou G. Y., Liu C. B., Tang Y. H., Luo S. L., Zeng Z. B., Liu Y. T., Xu R., Chu L. (2015). Chem. Eng. J..

[cit21] Hou W. J., Zhang Y. M., Liu T., Lu H. W., He L. (2015). RSC Adv..

[cit22] Hu X. J., Liu Y. G., Wang H., Chen A. W., Zeng G. M., Liu S. M., Guo Y. M., Hu X., Li T. T., Wang Y. Q., Zhou L., Liu S. H. (2013). Sep. Purif. Technol..

[cit23] Duru I., Ege D., Kamali A. R. (2016). J. Mater. Sci..

[cit24] Alagappan P. N., Heimann J., Morrow L., Andreoli E., Barron A. R. (2017). Sci. Rep..

[cit25] Zhao G. X., Ren X. M., Gao X., Tan X. L., Li J. X., Chen C. L., Huang Y. Y., Wang X. K. (2011). Dalton Trans..

[cit26] Liu P. Y., Yan T. T., Zhang J. P., Shi L. Y., Zhang D. S. (2017). J. Mater. Chem. A.

[cit27] Liu P. Y., Yan T. T., Shi L. Y., Park H. S., Chen X. C., Zhao Z. G., Zhang D. S. (2017). J. Mater. Chem. A.

[cit28] Wang M., Xu X. T., Tang J., Hou S. J., Hossain M. S. A., Pan L. K., Yamauchi Y. (2017). Chem. Commun..

[cit29] Chen M. M., Wei D., Chu W., Wang T., Tong D. G. (2017). J. Mater. Chem. A.

[cit30] Liu L. J., Guo X. R., Tallon R., Huang X. K., Chen J. H. (2017). Chem. Commun..

[cit31] Wang S., Li X., Liu Y., Zhang C., Tan X., Zeng G., Song B., Jiang L. (2017). J. Hazard. Mater..

[cit32] Novoselov K. S., Geim A. K., Morozov S. V., Jiang D., Zhang Y., Dubonos S. V., Grigorieva I. V., Firsov A. A. (2004). Science.

[cit33] Lerf A., He H. Y., Forster M., Klinowski J. (1998). J. Phys. Chem. B.

[cit34] Chang H., Wu H. (2013). Energy Environ. Sci..

[cit35] Zhao G., Wen T., Chen C., Wang X. (2012). RSC Adv..

[cit36] Zhao G., Li J., Ren X., Chen C., Wang X. (2011). Energy Environ. Sci..

[cit37] Rosillo-Lopez M., Lee T. J., Bella M., Hart M., Salzmann C. G. (2015). RSC Adv..

[cit38] Rosillo-Lopez M., Salzmann C. G. (2016). Carbon.

[cit39] Chen J., Yao B. W., Li C., Shi G. Q. (2013). Carbon.

[cit40] Ahmed M. J., Mamun M. A. (2001). Talanta.

[cit41] Boehm H. P. (2002). Carbon.

[cit42] Chiang T. C., Seitz F. (2001). Ann. Phys..

[cit43] Yumitori S. (2000). J. Mater. Sci..

[cit44] Zhao G. X., Li J. X., Ren X. M., Chen C. L., Wang X. K. (2011). Environ. Sci. Technol..

[cit45] Sitko R., Turek E., Zawisza B., Malicka E., Talik E., Heimann J., Gagor A., Feist B., Wrzalik R. (2013). Dalton Trans..

[cit46] Dimiev A. M., Alemany L. B., Tour J. M. (2013). ACS Nano.

[cit47] Dougherty D. A. (1996). Science.

[cit48] Jeong S. Y., Kim S. H., Han J. T., Jeong H. J., Lee G. W. (2012). Adv. Funct. Mater..

[cit49] Machida M., Mochimaru T., Tatsumoto H. (2006). Carbon.

[cit50] Burgess J. , Metal ions in solution, Ellis Horwood, Chinchester, England, 1978

[cit51] Pesek J. J., Schneider J. F. (1988). J. Inorg. Biochem..

[cit52] Sisombath N. S., Jalilehvand F., Schell A. C., Wu Q. (2014). Inorg. Chem..

[cit53] Park S., Lee K.-S., Bozoklu G., Cai W., Nguyen S. T., Ruoff R. S. (2008). ACS Nano.

[cit54] Yang S. T., Chang Y. L., Wang H. F., Liu G. B., Chen S., Wang Y. W., Liu Y. F., Cao A. N. (2010). J. Colloid Interface Sci..

