# Supplementary material for: Highly efficient heavy-metal extraction from water with carboxylated graphene nanoflakes
Source: RSC Adv. 2018 Mar 20;8(20):11043–50. doi: 10.1039/c8ra00823j (PMC9079067; doi:10.1039/c8ra00823j)
Supplement: RA-008-C8RA00823J-s001 [file RA-008-C8RA00823J-s001.pdf]

Electronic Supplementary Material (ESI) for RSC Advances.
This journal is © The Royal Society of Chemistry 2018

Highly efficient heavy-metal extraction from water with carboxylated graphene nanoflakes

Martin Rosillo-Lopez and Christoph G. Salzmann*

Department of Chemistry, University College London, 20 Gordon Street, London WC1H 0AJ, United Kingdom.

* Corresponding author. e-mail: c.salzmann@ucl.ac.uk (Christoph G. Salzmann), tel.: +44 20 7679 8864.

Electronic Supplementary Information

Contents

1. Preparation of carbon materials 2
 1.1. Preparation of cx-GNF[1] 2
 1.2. Preparation of nGO[2] 2
 1.3. Preparation of GO[3, 4] 2
2. XPS survey spectra of $M^{2+/3+}$@carbon 3
3. Kinetic study of Pb^{2+} adsorption onto the carbon materials 4
4. XPS C1s spectra of $M^{2+/3+}$@nGO 4
5. ATR-IR spectra of $M^{2+/3+}$@nGO and $M^{2+/3+}$@GO 5
6. XPS survey spectra of cx-GNF treated with Pb^{2+} in excess of Ca^{2+} or Mg^{2+} cations 6
7. XPS survey spectra of cx-GNF treated with 5 mM Pb^{2+} in commercial drinking water 7
8. Calibration curves of DMTD-K^+ treated with $Pb(NO_3)_2$ 7
9. XPS Survey and C1s regions of Pb^{2+}@carbon treated with 0.005 M $Pb(NO_3)_2$ 8
10. Optical absorbance spectra of the filtrates of cx-GNF, nGO and GO after treatment with 5 mM $Pb(NO_3)_2$ 9
11. XPS Survey and C1s regions of Pb^{2+}@AC treated with 0.1 M $Pb(NO_3)_2$ 9
12. References 10

1. Preparation of carbon materials

1.1. Preparation of cx-GNF[1]

1.00 g of MWCNT (3 to 15 walls, 5-20 nm outer diameter and 2-6 nm inner diameter and 1 to 10 μm in length; purchased from Bayer Materials Science) were ultrasonicated in a 100 mL mixture of 3:1 vol% conc. sulfuric acid (95-97% w/w) and conc. nitric acid (70% w/w) for 30 minutes. The reaction mixture was heated for 2 h at 100°C, cooled to room temperature and diluted three-fold with deionised water. The black dispersion was filtered through a 200 nm track-etched polycarbonate membrane and the black residue on the membrane was discarded. The black filtrate was neutralised with KOH and the white salt precipitate (consisting mainly of K_2SO_4) was removed by filtration. The black filtrate was reacidified with dilute formic acid and then dialysed against high-purity *Milli-Q* water using a SpectraPor 3 regenerated cellulose dialysis membrane (Spectrum laboratories, MWCO 3.5 kDa). Once the conductivity of the surrounding water was <5 μS cm^{-1} the dispersion was passed over a cation exchange resin (Amberlite IR120, Sigma-Aldrich), dialysed once more and freeze dried to give 160 mg of brown-black cx-GNFs.

1.2. Preparation of nGO[2]

Arc-discharge (ADC) material consisting of single-wall carbon nanotubes (SWCNT) (diameter: 0.7- 1.2 nm, length: 10-50 μm), multi-wall carbon nanotubes (MWCNT) (diameter: 8-20 nm, length: 2-20 μm) and graphitic carbon was purchased from the Materials and Electrochemical Research (MER) Corporation (MRSW grade). 1.00 g of this material was ultrasonicated in 50 mL of 9 M nitric acid for 30 min followed by refluxing in air for 20 h. The resulting black dispersion was diluted three-fold with deionised water and then filtered under vacuum through a 200 nm track-etched Whatman polycarbonate membrane. The black residue on the membrane was discarded and the black filtrate was neutralised by the addition of NaOH pellets. On neutralisation, precipitation of the sodium salt of nGO (Na^+@nGO) occurred. The mixture was then filtered under vacuum once again and the filtrate (containing $NaNO_3$) was discarded. The Na^+@nGO left on the membrane was re-dispersed in dilute nitric acid and dialysed against high-purity *Milli-Q* water water *via* a SpectraPor 3 regenerated cellulose dialysis membrane (Spectrum laboratories, MWCO 3.5 kDa). The dialysis was considered complete when the conductivity of the surrounding water was <5 μS cm^{-1}. The purified dispersion was freeze dried to obtain the black nGO material (210 mg).

1.3. Preparation of GO[3, 4]

Graphite flakes (500 mg, 100 mesh, Sigma Aldrich) was combined with concentrated sulfuric acid (12.5 mL, 97% w/w) at 0°C with stirring. Potassium permanganate (1.50 g) was added slowly to the reaction mixture and the mixture was heated at 40°C for 30 min. Deionised water (25 mL) was added carefully to the mixture and the mixture was heated once more at 95°C for

15 min with stirring. The resulting brown mixture was then diluted by addition of deionised water (85 mL) followed by dropwise addition of hydrogen peroxide (5 ml, 30% v/v) to reduce any excess permanganate. The resulting yellow-green mixture was filtered through a 200 nm polycarbonate membrane, washed with aqueous HCl (75 mL, 10% v/v) and allowed to dry. The dry powder was re-dispersed in deionised water (100 mL) and dialysed against deionised water using a SpectraPor 3 regenerated cellulose dialysis membrane (Spectrum Laboratories, MWCO 3.5 kDa). The GO was then exfoliated by ultrasonication for 90 minutes, and the dispersion was centrifuged at 3000 rpm for 40 min. The supernatant was collected and ultrasonicated once more for 30 min, filtered through compacted glass wool, and passed over a cation exchange resin (Amberlite IR120, Sigma-Aldrich). The dispersion was concentrated and freeze dried to obtain a light brown solid (500 mg).

2. XPS survey spectra of $M^{2+/3+}$@carbon

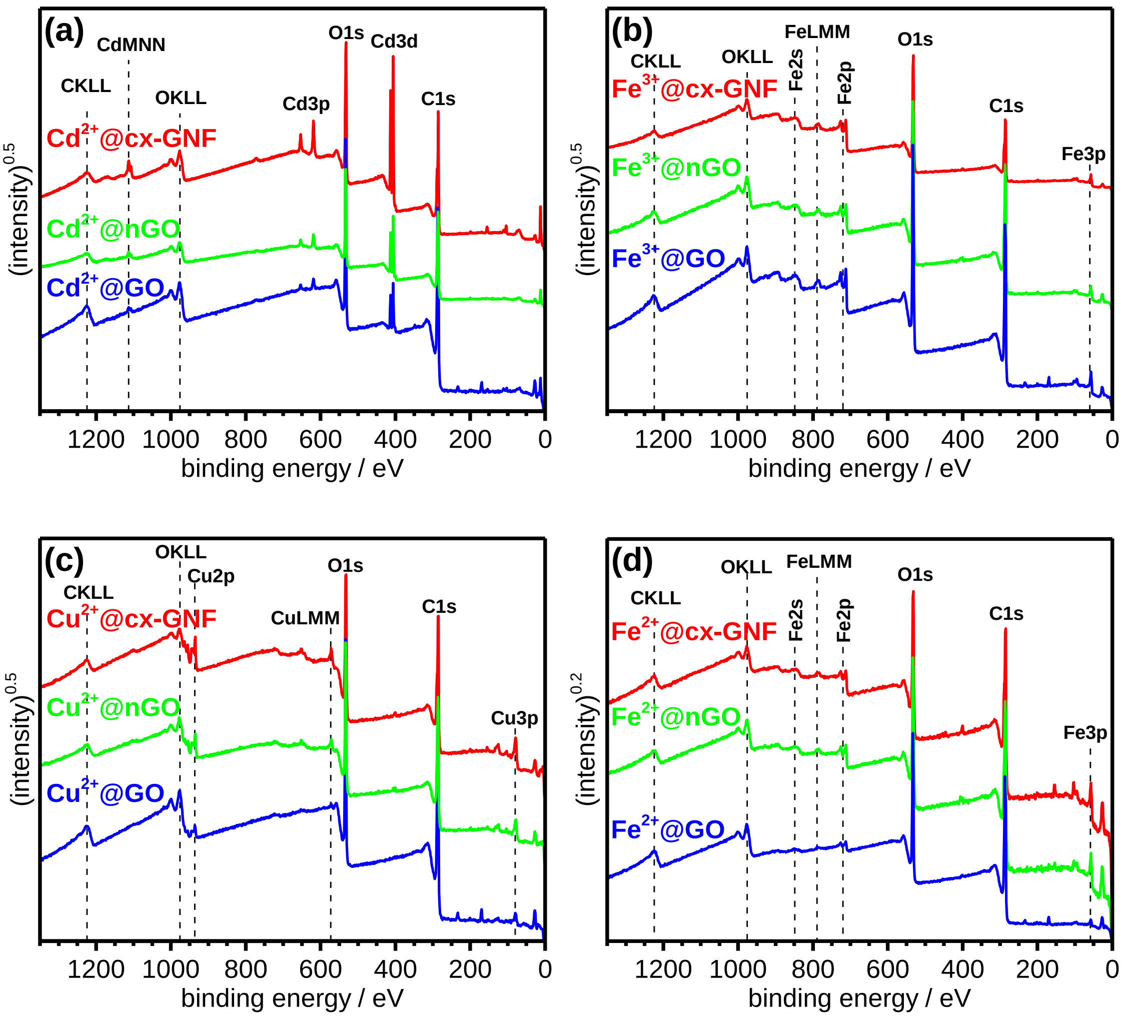

Fig. S1. *The XPS survey spectra of $M^{2+/3+}$@cx-GNF (red), $M^{2+/3+}$@nGO (green) and $M^{2+/3+}$@GO (blue) treated with Cd^{2+}, Fe^{3+}, Cu^{2+} and Fe^{2+} cations are shown in (a)-(d) respectively. Black dashed lines indicate the major peaks in the spectra.*

3. Kinetic study of Pb^{2+} adsorption onto the carbon materials

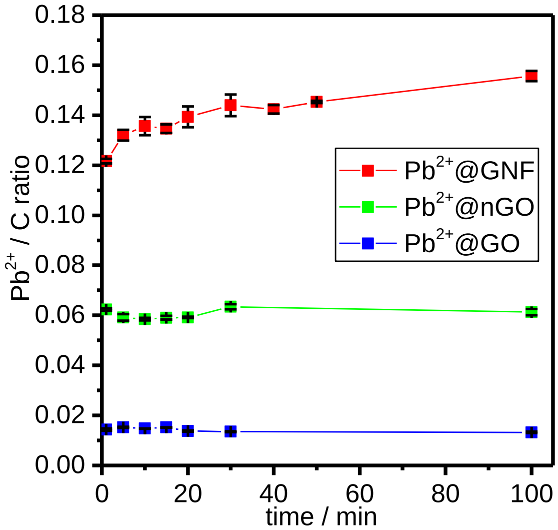

Fig. S2. *Influence of the exposure time of cx-GNF (red), nGO (green) and GO (blue) to 0.1 M $Pb(NO_3)_2$ solutions on the Pb^{2+}/C atomic ratio obtained from XPS. [Conditions: 10 mg of each carbon material was treated with 10 mL of 0.1 M $Pb(NO_3)_2$, in the same way as carried out in Fig. 2(b)].*

4. XPS C1s spectra of $M^{2+/3+}$@nGO

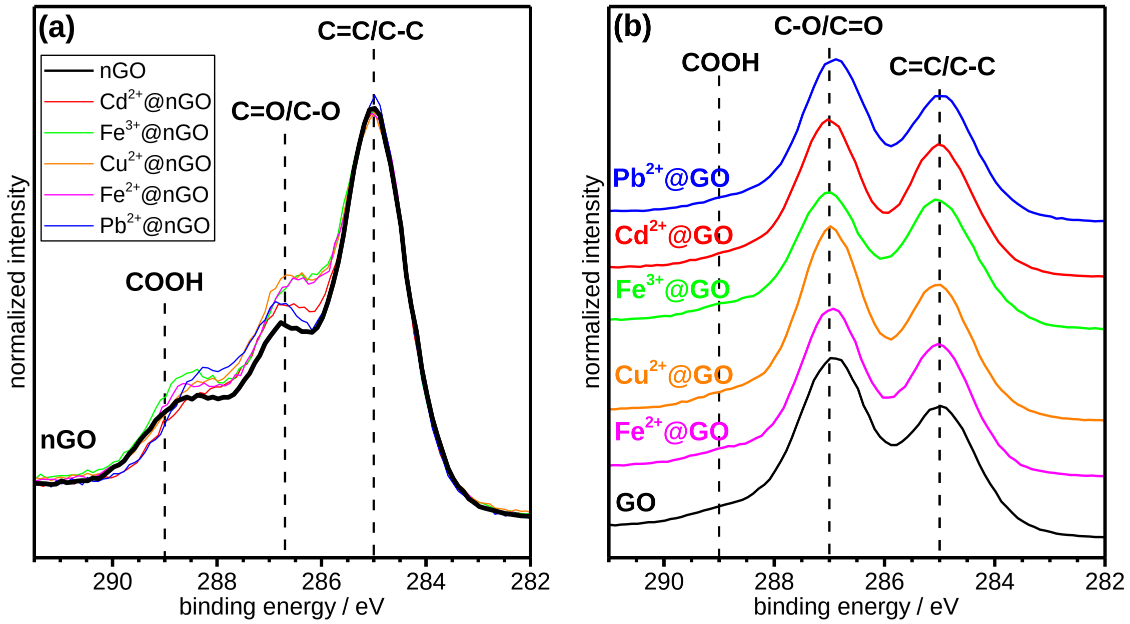

Fig. S3. *XPS C1s spectra of (a) nGO and (b) GO treated with Fe^{2+} cations (magenta), Cu^{2+} cations (orange), Fe^{3+} cations (green), Cd^{2+} cations (red), Pb^{2+} cations (blue) and controls (black).*

5. ATR-IR spectra of $M^{2+/3+}$@nGO and $M^{2+/3+}$@GO

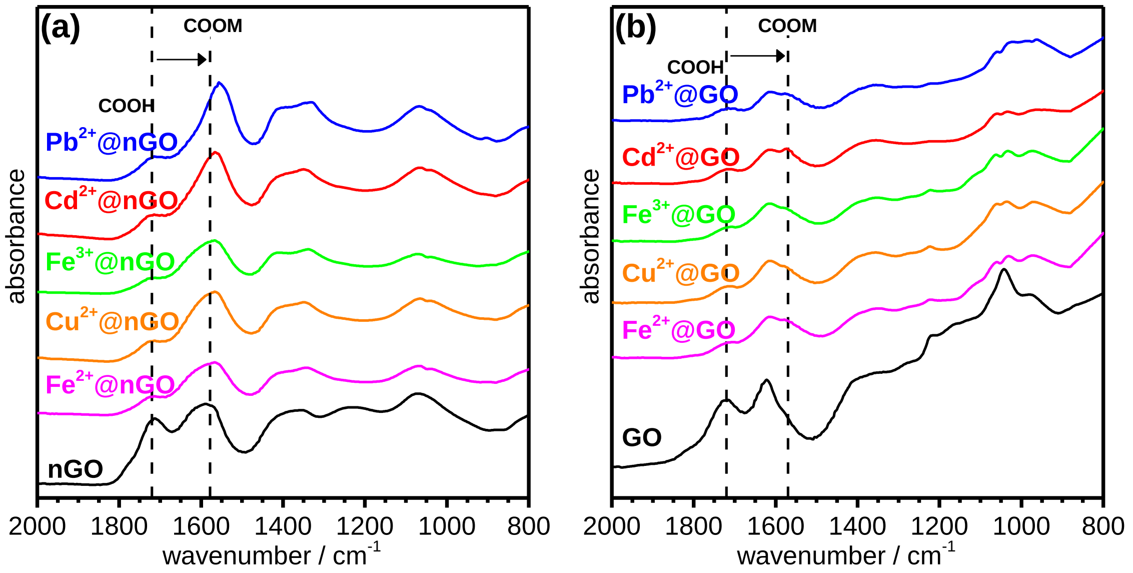

Fig. S4. *ATR-IR spectra of (a) nGO and (b) GO treated with Fe^{2+} cations (pink), Cu^{2+} cations (orange), Fe^{3+} cations (green), Cd^{2+} cations (red), Pb^{2+} cations (blue) and controls (black).*

6. XPS survey spectra of cx-GNF treated with Pb^{2+} in excess of Ca^{2+} or Mg^{2+} cations

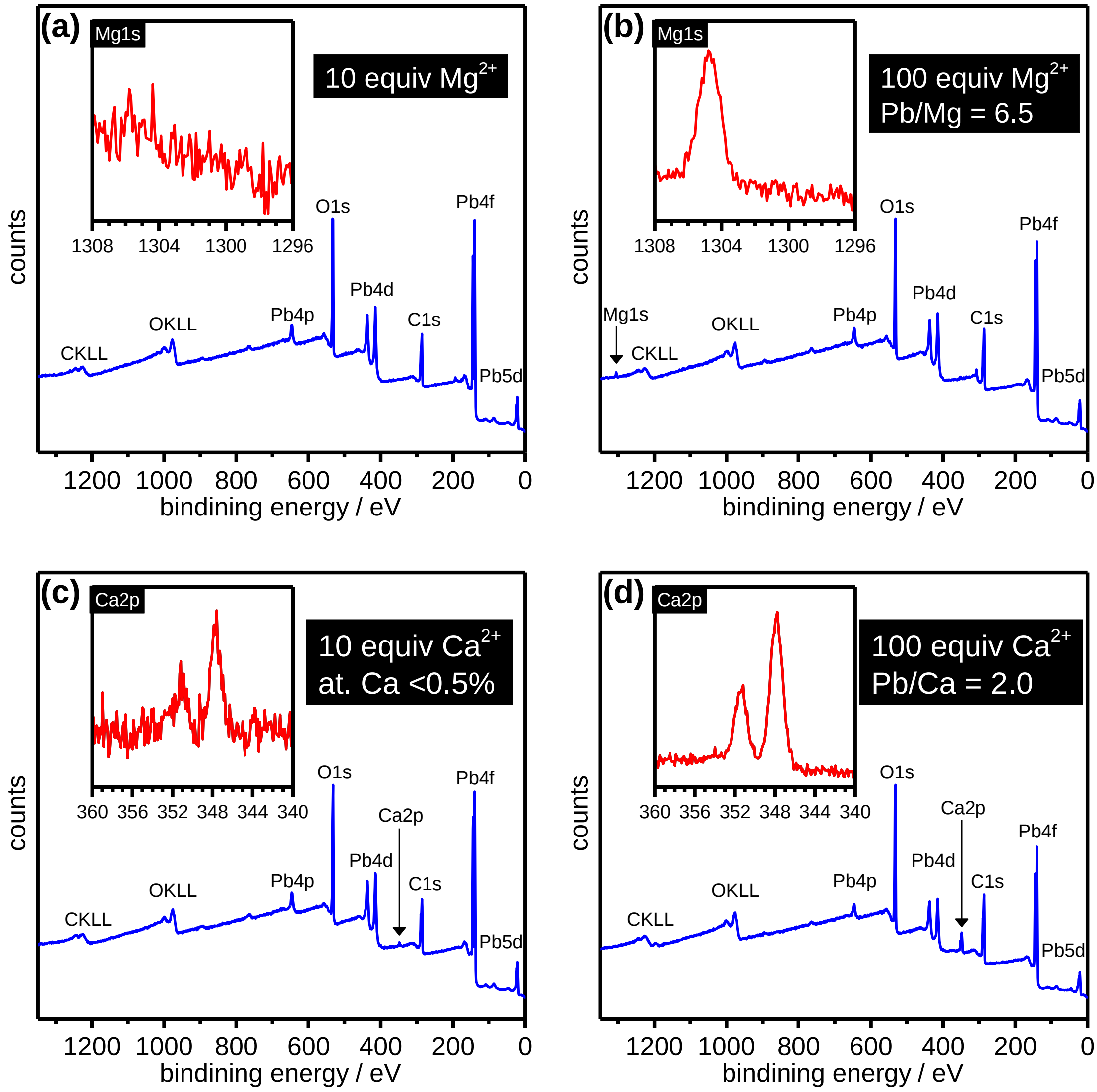

Fig. S5. *XPS survey spectra (outset) and Mg1s or Ca2p regions (inset) of cx-GNF treated with a mixture of* Pb^{2+} *cations and 10 equiv.* Mg^{2+} *(a) or* Ca^{2+}*(b) or 100 equiv.* Mg^{2+} *(c) or* Ca^{2+} *(d).*

7. XPS survey spectra of cx-GNF treated with 5 mM Pb^{2+} in commercial drinking water

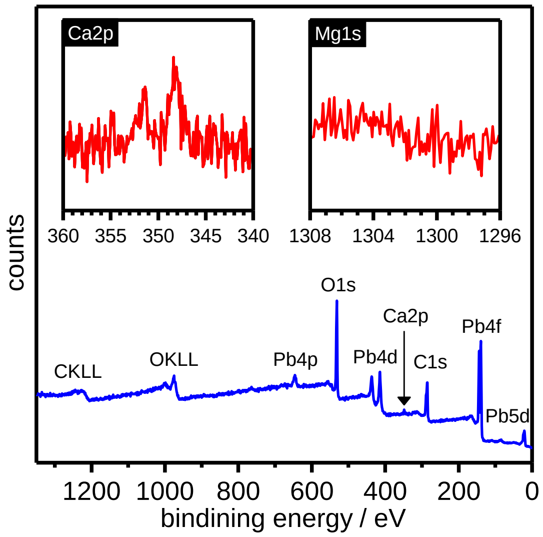

Fig. S6. *XPS survey spectrum (outset) as well as Ca2p and Mg1s regions (insets) of cx-GNF treated with 5 mM $Pb(NO_3)_2$ in commercial drinking water.*

8. Calibration curves of DMTD-K^+ treated with $Pb(NO_3)_2$

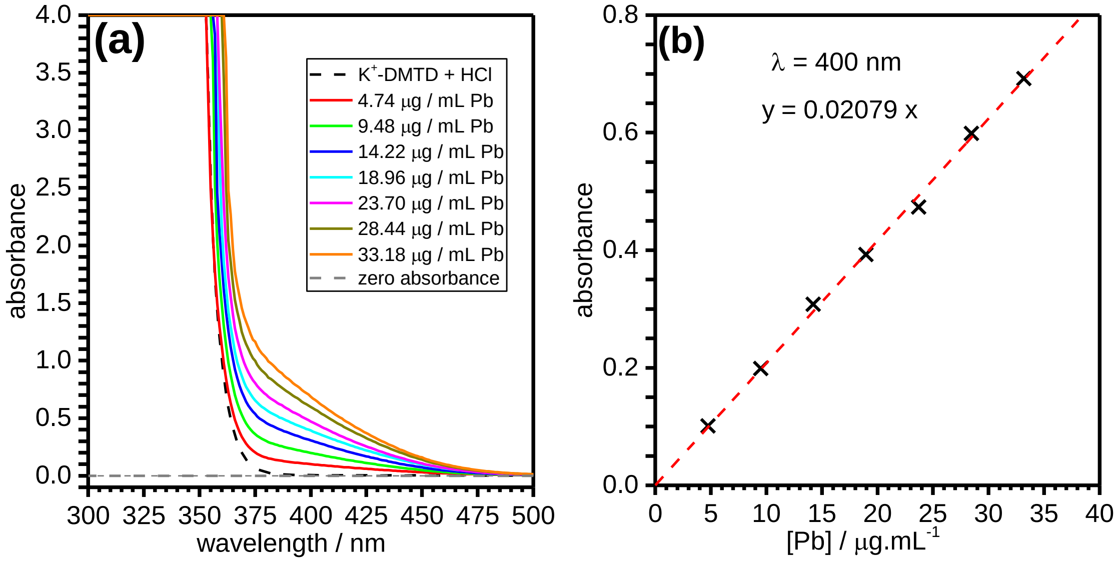

Fig. S7. *(a) Optical absorbance spectra in the λ = 500 to 300 nm range of DMTD-K^+ treated with Pb^{2+}concentrations indicated in the legend and (b) absorbance values at λ = 400 nm plotted against Pb^{2+}concentration showing that the Beer-Lambert law is obeyed.*

9. XPS Survey and C1s regions of Pb^{2+}@carbon treated with 0.005 M $Pb(NO_3)_2$

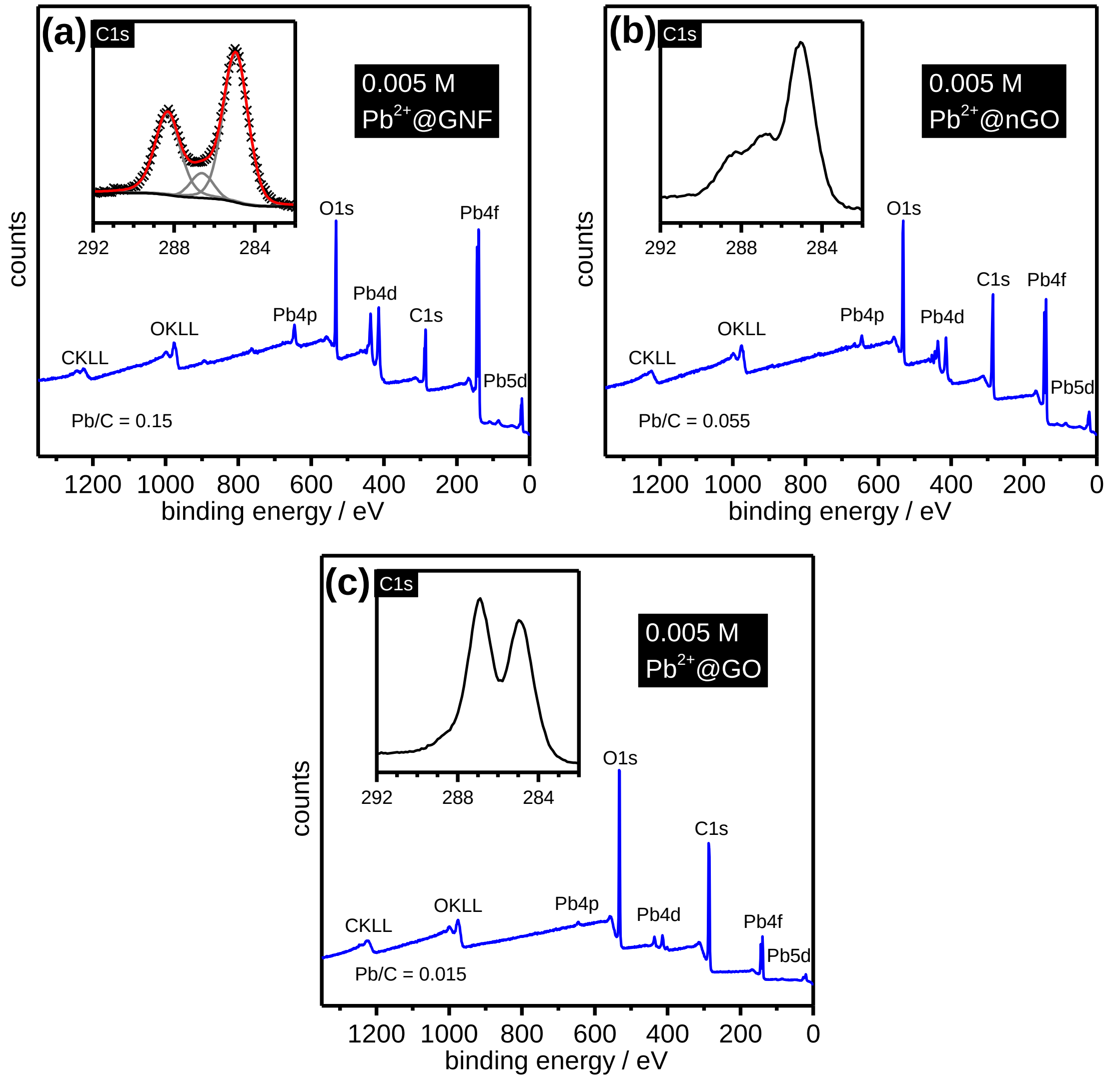

Fig. S8. *XPS survey spectra (outset) and C1s region (inset) of Pb^{2+}@carbon treated with 0.005 M $Pb(NO_3)_2$.*

10. Optical absorbance spectra of the filtrates of cx-GNF, nGO and GO after treatment with 5 mM $Pb(NO_3)_2$

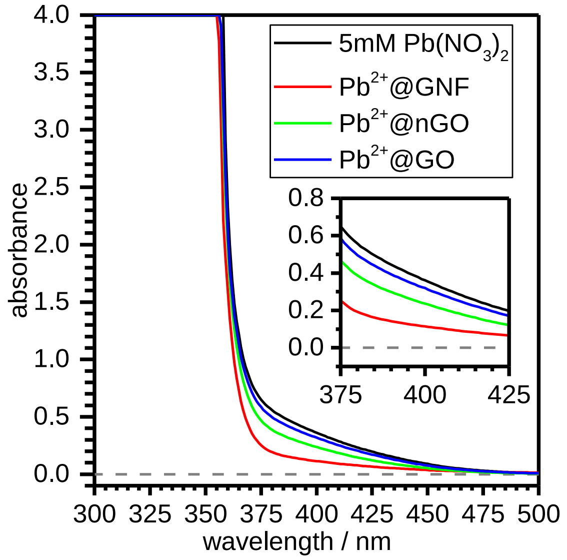

Fig. S9. *Optical absorbance spectra in the λ = 500-300 nm range (outset) and zoomed in region at λ = 400 nm (inset) of the remaining (unreacted) Pb^{2+} cations (i.e. the filtrate) after treatment of 5 mM $Pb(NO_3)_2$ with the cx-GNFs, nGO and GO.*

11. XPS Survey and C1s regions of Pb^{2+}@AC treated with 0.1 M $Pb(NO_3)_2$

AC denotes activated charcoal as described in the main article.

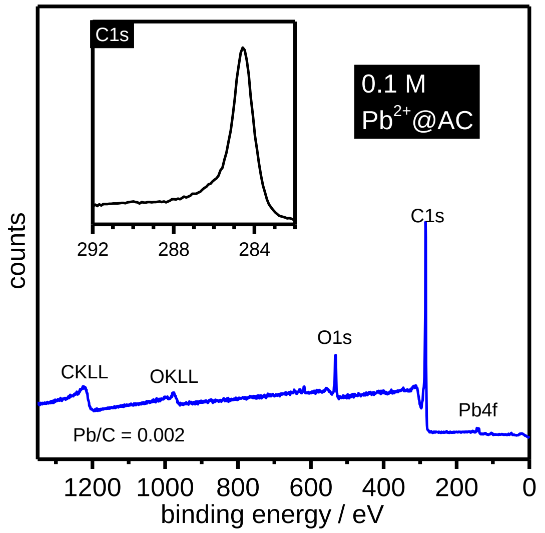

Fig. S10. *XPS survey spectra (outset) and C1s region (inset) of Pb^{2+}@AC treated with 0.1 M $Pb(NO_3)_2$.*

12. References

1. M. Rosillo-Lopez, T. J. Lee, M. Bella, M. Hart and C. G. Salzmann, *RSC Adv.*, 2015, **5**, 104198-104202.
2. M. Rosillo-Lopez and C. G. Salzmann, *Carbon*, 2016, **106**, 56-63.
3. J. Chen, B. W. Yao, C. Li and G. Q. Shi, *Carbon*, 2013, **64**, 225-229.
4. W. S. Hummers and R. E. Offeman, *J. Am. Chem. Soc.*, 1958, **80**, 1339-1339.
